# Association between chronic kidney disease and open-angle glaucoma in South Korea: a 12-year nationwide retrospective cohort study

**DOI:** 10.1038/s41598-022-07190-8

**Published:** 2022-03-01

**Authors:** Jun-Soo Ro, Jong Youn Moon, Tae Kwann Park, Si Hyung Lee

**Affiliations:** 1 Department of Health Policy and Management, College of Medicine, Seoul National University, Seoul, Republic of Korea; 2 Center for Public Healthcare, Gil Medical Center, Gachon University, Incheon, Republic of Korea; 3 Department of Ophthalmology, College of Medicine, Soonchunhyang University, Cheonan, Republic of Korea; 4 Department of Ophthalmology, Soonchunhyang University Hospital Bucheon, 170 Jomaru-ro, Wonmi-gu, Bucheon, 14584 Republic of Korea

**Keywords:** Epidemiology, Glaucoma, Chronic kidney disease

## Abstract

Various non-intraocular pressure factors have been identified as possible risk factors for open-angle glaucoma (OAG). However, there is still controversy around the association between OAG and chronic kidney disease (CKD). In this study, we used a nationwide cohort to investigate the risk of OAG in the 12 years following a diagnosis of CKD. This retrospective cohort study included 1,103,302 subjects from the Korean National Health Insurance Service National Sample Cohort database. The CKD group (n = 1318) included patients who were initially diagnosed with CKD between 2003 and 2008. The subjects in the comparison group were matched at a 1:5 ratio using propensity scores. In multivariate Cox regression analysis, a diagnosis of CKD was significantly associated with an increased incidence of OAG (hazard ratio [HR] = 1.546, 95% confidence interval [CI] 1.363–1.754, *p* < 0.001). Further analysis revealed that the risk of OAG increased with the severity of CKD (mild to moderate CKD [CKD stage 1–3]: HR = 1.280, 95% CI 1.077–1.521, *p* = 0.005; advanced CKD [CKD stage 4–5]: HR = 1.861, 95% CI 1.589–2.180, *p* < 0.001). In subgroup analysis, female CKD patients had a greater risk of developing OAG than males, and subjects with CKD aged ≥ 40 years were more likely to develop OAG compared with those aged < 40 years. Our study demonstrates that CKD is a significant risk factor for OAG and that severe CKD is associated with an increased risk of developing OAG.

## Introduction

Open-angle glaucoma (OAG), one of the leading causes of irreversible blindness, is characterized by chronic progressive glaucomatous optic neuropathy with corresponding visual field defects^1,2^. While intraocular pressure (IOP) is known to be the main cause of OAG development and progression, several non-IOP factors are risk factors for OAG^3–6^. Vascular risk factors are among those suggested as a major cause of glaucomatous damage, which would support vascular and ischemic mechanisms of OAG^6–8^. However, there is still controversy around the role of these vascular factors. Studies of vascular diseases have had contradictory results.

Chronic kidney disease (CKD) is a major microvascular disease involving renal function impairment and is associated with various cardiovascular and metabolic comorbidities^9,10^. Its global prevalence is increasing, and it has become a serious public health problem worldwide^11,12^. There have been reports of associations between CKD and vision-threatening ocular conditions, including diabetic retinopathy^13,14^, cataracts^13,15^, age-related macular degeneration^16–18^, and OAG. Especially, CKD and OAG have been reported to have common risk factors, including hypertension^19^, diabetes mellitus^9^, hyperlipidemia^20^, and old age^19^, and may share similar pathogenetic mechanisms such as atherosclerosis^21,22^, oxidative stress^23,24^, and renin-angiotensin system (RAS) dysfunction^25^. Moreover, several population-based studies have shown a positive association between CKD and OAG^26–30^, indicating both an increased risk of OAG in CKD patients as well as an increased risk of CKD in OAG patients. On the other hands, other studies have not found a significant association between the two conditions^13,31,32^. This leaves a room for an opportunity to explore the nature of the causal relationship between the two conditions further using longitudinal study design.

In light of above, in this study, we investigated the risk of developing OAG after CKD diagnosis using a representative sample of approximately 1.1 million South Koreans from the National Health Insurance Service-National Sample Cohort 2002–2015 (NHIS-NSC 2002–2015). In addition, we analyzed the risk of developing OAG according to CKD severity.

## Results

Table 1 shows the baseline characteristics of the study population and the differences between the OAG group and the control group. There was a significant difference in the prevalence of CKD in the OAG group and the control group (*p* < 0.001). Subjects in the OAG group were older (*p* < 0.001), had lower income (*p* < 0.001), were more likely to live in rural areas (*p* < 0.001), and had lower CCI scores (*p* < 0.001). Hypertension (*p* < 0.001), diabetes mellitus (*p* < 0.001), and hyperlipidemia (*p* < 0.001) were more prevalent in the OAG group than in the control group. There were no significant differences in sex or ischemic stroke.

**Table 1:** Demographic characteristics of study population.

	OAG (n = 1318, column %)	No OAG (n = 24,242, column %)	*p* value
**CKD**			
No	972 (73.7)	20,328 (83.9)	< 0.001*
Yes	346 (26.3)	3914 (16.1)	
**Age**			
< 50	75 (5.7)	4155 (17.1)	< 0.001*
50–59	141 (10.7)	4485 (18.5)	
60–69	293 (22.2)	5665 (23.4)	
70–79	514 (39.0)	6530 (26.9)	
> 80	295 (22.4)	3407 (14.1)	
**Sex**			
Male	798 (60.5)	14,742 (60.8)	0.848
Female	520 (39.5)	9500 (39.2)	
**Income**			
70–100 percentile (Low)	840 (63.8)	13,020 (53.7)	< 0.001*
40–70 percentile	239 (18.1)	5497 (22.7)	
> 40 percentile (High)	239 (18.1)	5725 (23.6)	
**City**			
City resident	685 (52.0)	11,243 (46.4)	< 0.001*
Rural resident	633 (48.0)	12,999 (53.6)	
**CCI**			
< 3	222 (16.8)	7242 (29.9)	< 0.001*
≥ 3	1096 (83.2)	17,000 (70.1)	
**Diabetes mellitus**			
No	260 (19.7)	9623 (39.7)	< 0.001*
Yes	1058 (80.3)	14,619 (60.3)	
**Hypertension**			
No	236 (17.9)	8716 (36.0)	< 0.001*
Yes	1082 (82.1)	15,526 (64.0)	
**Hyperlipidemia**			
No	372 (28.2)	11,259 (46.4)	< 0.001*
Yes	946 (71.8)	12,983 (53.6)	
**Stroke**			
No	1278 (97)	23,686 (97.7)	0.082
Yes	40 (3.0)	556 (2.3)	

*p < 0.05.

*OAG*, open-angle glaucoma; *CKD*, chronic kidney disease; *CCI*, Charlson comorbidity index.

The risk of a CKD patient developing OAG during the 12-year follow-up period was analyzed using multivariate Cox hazard regression analysis. The risk of developing OAG during the 12-year follow-up period was significantly higher in the CKD group than the control group (CKD group: adjusted HR = 1.546, 95% CI 1.363–1.754, *p* < 0.001) (Table 2). Higher CKD stages were associated with a higher risk of OAG (CKD group 1: adjusted HR = 1.280, 95% CI 1.077–1.521, *p* = 0.005; CKD group 2: adjusted HR = 1.861, 95% CI 1.589–2.180, *p* < 0.001) (Table 3). Older age, low income, rural residency, hypertension, diabetes mellitus, and hyperlipidemia were also associated with an increased risk of OAG.

**Table 2:** Hazard ratios for open-angle glaucoma in multivariable Cox regression analysis.

	Hazard ratio*	95% CI	*p* value
**CKD**			
No CKD	–	–	–
CKD	1.546	1.363–1.754	< 0.001*
**Age**			
< 50	0.361	0.274–0.475	< 0.001*
50–59	0.460	0.372–0.567	< 0.001*
60–69	0.653	0.553–0.771	< 0.001*
70–79	0.900	0.779–1.040	0.155
> 80	–	–	–
**Sex**			
Male	–	–	–
Female	1.071	0.958–1.197	0.227
**Income**			
70–100 percentile (Low)	–	–	–
40–70 percentile	0.758	0.656–0.875	< 0.001*
> 40 percentile (High)	0.749	0.648–0.866	< 0.001*
**Residential area**			
Urban	–	–	–
Rural	1.227	1.101–1.367	< 0.001*
**CCI**			
< 3	1.325	0.996–1.762	0.053
≥3	–	–	–
**Diabetes mellitus**			
No	–	–	–
Yes	2.280	1.736–2.995	< 0.001*
**Hypertension**			
No	–	–	–
Yes	1.236	1.052–1.453	0.01*
**Hyperlipidemia**			
No	–	–	–
Yes	1.482	1.303–1.685	< 0.001*
**Ischemic stroke**			
No	–	–	–
Yes	1.351	0.978–1.867	0.068

*p < 0.05.

*CKD*, chronic kidney disease; *CCI*, Charlson comorbidity index.

*Adjusted for all variables shown in the table.

**Table 3:** Hazard ratios for open-angle glaucoma in multivariable Cox regression analysis according to severity of chronic kidney disease.

	Hazard ratio*	95% CI	*p* value
**CKD**			
No CKD	–	–	–
CKD (Stage 1–3)	1.280	1.077–1.521	0.005*
CKD (Stage 4–5)	1.861	1.589–2.180	< 0.001*
**Age**			
< 50	0.352	0.267–0.463	< 0.001*
50–59	0.452	0.366–0.557	< 0.001*
60–69	0.647	0.548–0.764	< 0.001*
70–79	0.900	0.778–1.040	0.151
> 80	–	–	–
**Sex**			
Male	–	–	–
Female	1.081	0.967–1.208	0.1711
**Income**			
70–100 percentile (Low)	–	–	–
40–70 percentile	0.755	0.654–0.872	< 0.001*
> 40 percentile (High)	0.750	0.648–0.866	< 0.001*
**Residential area**			
Urban	–	–	–
Rural	1.234	1.107–1.375	< 0.001*
**CCI**			
< 3	1.320	0.998–1.745	0.051
≥ 3	–	–	–
**Diabetes mellitus**			
No	–	–	–
Yes	2.250	1.722–2.941	< 0.001*
**Hypertension**			
No	–	–	–
Yes	1.220	1.038–1.434	0.016*
**Hyperlipidemia**			
No	–	–	–
Yes	1.492	1.312–1.697	< 0.001*
**Ischemic stroke**			
No	–	–	–
Yes	1.336	0.966–1.847	0.080

*p < 0.05.

*OAG*, open-angle glaucoma; *CKD*, chronic kidney disease; *CCI*, Charlson comorbidity index.

*Adjusted for all variables shown in the table.

**Figure 1:**
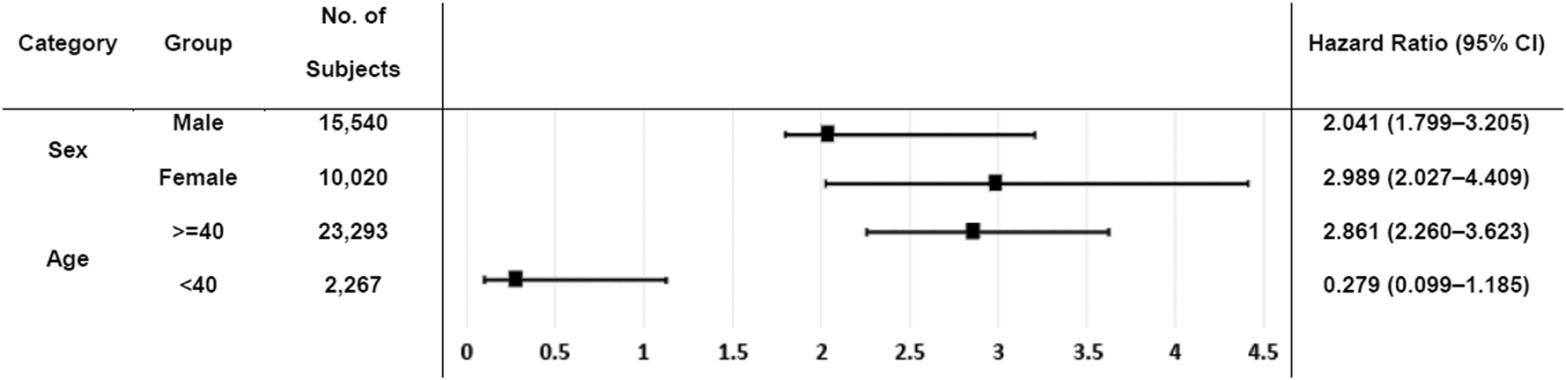
Subgroup analysis of developing open-angle glaucoma according to age and sex based on multivariate Cox regression.

In subgroup analyses based on multivariate Cox regression, the adjusted HR of OAG in the female CKD patients was higher (adjusted HR = 2.989) than that in the male CKD patients (adjusted HR = 2.041, Fig. 1). Moreover, the risk of developing AD in patients with OAG was higher in the subgroup of age ≥ 40 years (HR = 2.861) than that in the subgroup of age < 40 years (HR = 0.279).

**Figure 2:**
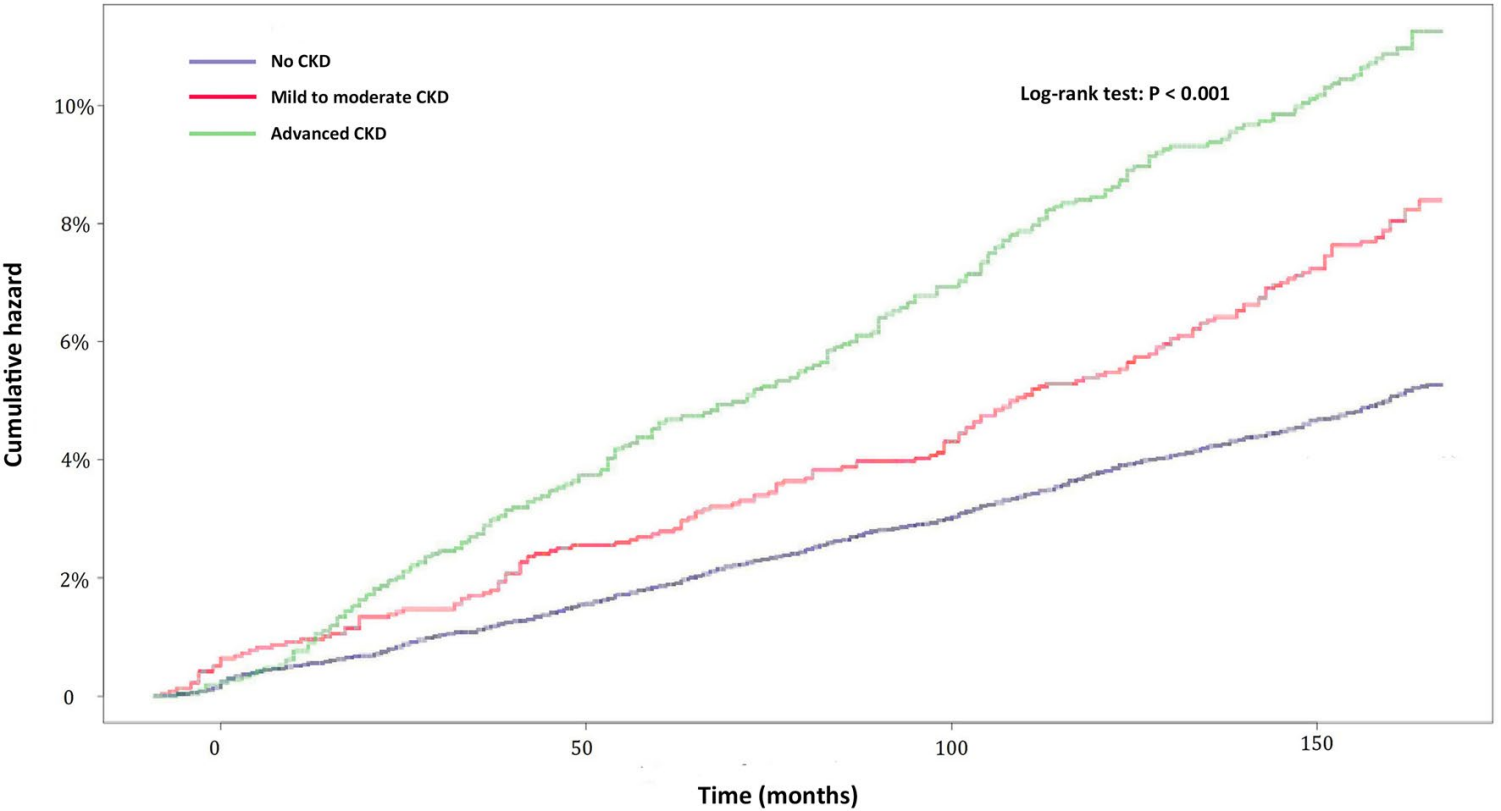
Cumulative hazards of open-angle glaucoma in CKD patients with stage 1–2 (mild to moderate CKD) and stage 3–5 (advanced CKD) by month.

**Figure 3:**
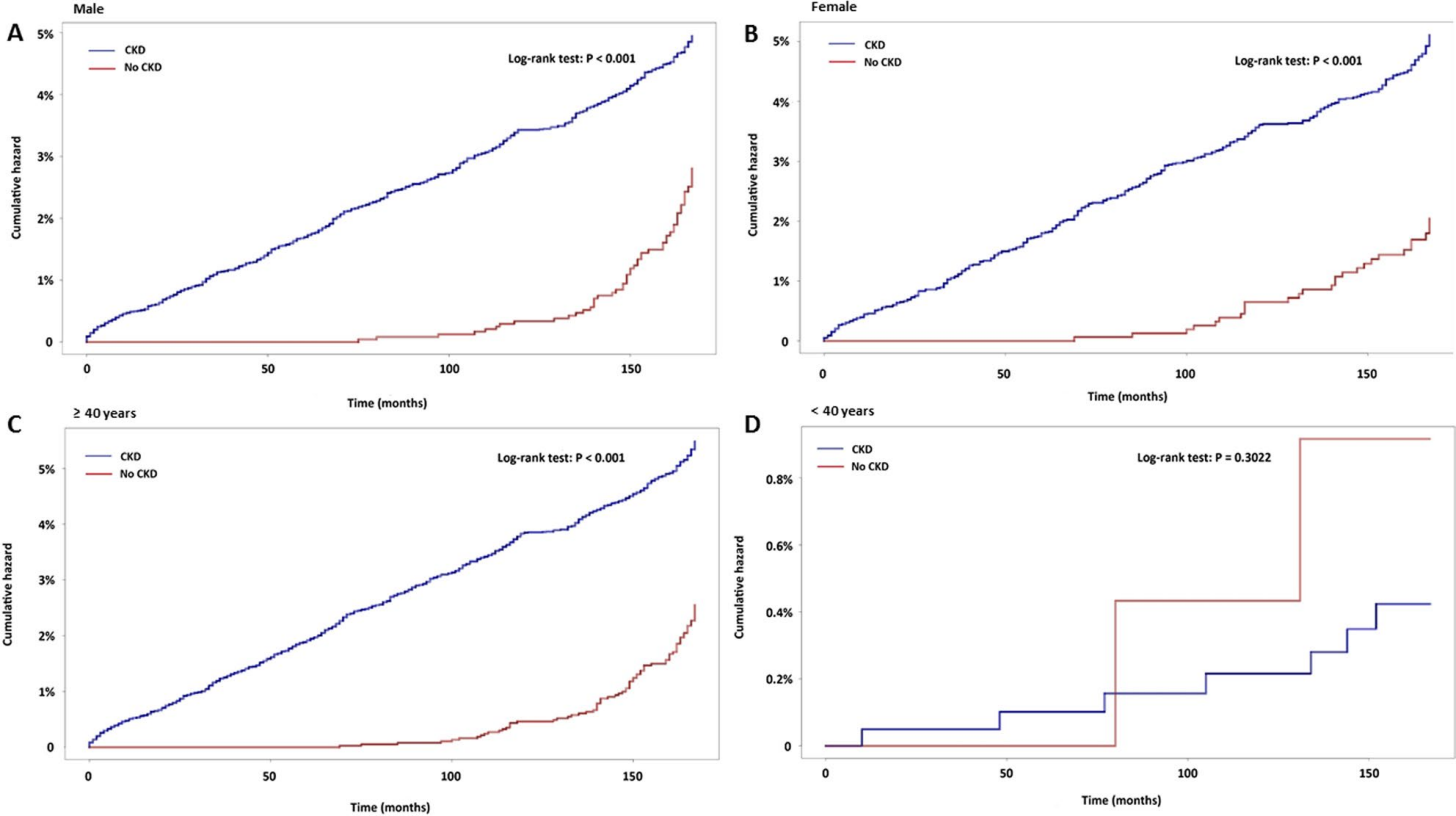
Cumulative hazards of open-angle glaucoma in CKD patients according to sex (**A,B**) and age group (≥ 40 or < 40 years, **C,D**).

As shown in the Kaplan–Meier survival curves in Fig. 1, the risk of OAG was higher for patients at CKD stages 1–3 (mild to moderate CKD) and CKD stages 4–5 (advanced CKD) than in the non-CKD comparison group (log-rank test, < 0.001) (Fig. 2). In addition, the cumulative incidence of OAG during the follow-up period was significantly higher in advanced CKD than mild to moderate CKD (log-rank test, *p* < 0.001). Furthermore, the risk of developing OAG was higher for CKD patients in both male and female subgroups, as well as in the subgroup of age ≥ 40 years (log-rank test, < 0.001, Fig. 3A–C). However, no significant difference in the cumulative OAG incidence was observed between the CKD and comparison group in the subgroup of age < 40 years (log-rank test, *p* = 0.3022, Fig. 3D).

## Discussion

In this population-based cohort study, we evaluated the risk of OAG in CKD patients using propensity score matching. We used multivariate Cox proportional hazard regression analysis and found that CKD was a significant risk factor for OAG. In a subgroup analysis in which CKD patients were divided into two groups based on CKD severity, there was an increased risk of OAG in CKD patients with more advanced disease. To our knowledge, this is the first study using a large longitudinal cohort from a nationwide database to evaluate the risk of OAG in CKD patients by disease stage and to show a significant increase in OAG risk with worsening kidney function.

Several population-based studies have explored the relationship between CKD and OAG, with conflicting results. A study using National Health and Nutrition Examination Survey data found no significant association between CKD and glaucoma^33^. Another report on a large pool of Asian population-based studies showed that neither lower estimated glomerular filtration rate (eGFR) nor CKD were associated with primary OAG (POAG) prevalence^31^. However, subgroup analysis revealed a significant association between lower eGFR and POAG prevalence in East Asians, including Korean and Chinese individuals^31^. This association was supported by a recent population-based cross-sectional study from South Korea demonstrating a positive association between lower eGFR and POAG^34^, suggesting that kidney function decline is a risk factor for POAG. The discrepancies in study results may be due to ethnic differences and the higher prevalence of normal-tension glaucoma (NTG) in East Asian countries. Vascular risk factors, including hypertension, diabetes mellitus, and hyperlipidemia, are considered more important in NTG than POAG^6^. Our findings, which are based on a longitudinal cohort study, are consistent with previous results demonstrating a causal relationship between CKD and OAG.

It has been suggested that CKD and OAG share several pathophysiological mechanisms. The most frequently mentioned mechanism is the RAS. The RAS is a systemic mechanism that is essential for maintaining blood pressure and electrolyte homeostasis, and has been reported to be upregulated in CKD patients^35^. In the eye, the RAS has a role in the production of aqueous humor from the ciliary body and its outflow through the trabecular meshwork^36,37^. Especially, Angiotensin II (AngII), a major active component of RAS system affected by the release of renin, has been reported to increase production and decrease outflow of AH by acting on non-pigmented epithelium of ciliary body as well as trabecular meshwork^38–40^. Another in vivo study using rabbits showed that intracamerally intected AngII diminished uveoscleral outflow^41^, one of main AH outflow pathway other than trabecular meshwork. Such changes in the eye may result in increased IOP, which is a well-known risk factor for the pathogenesis of glaucoma. Moreover, AngII also has been reported to play a significant role in the regulation of ophthalmic microcirculation by controlling contraction and relaxation of vascular endothelium^42,43^. Disruption in blood–brain barrier of the optic nerve head in glaucoma, as previously reported^44^, may result in local passage of systemic RAS component to the optic nerve and retina, leading to dysregulation of the retinal microcirculation. In addition, an animal study using a rodent model of glaucoma found that angiotensin-converting enzyme inhibitors and angiotensin receptor antagonists may have a beneficial effect on RGC survival^45^, suggesting that RAS may modulate RGC survival through exerting a neuroprotective effect.

Other possible common pathologic mechanisms between CKD and OAG may include atherosclerosis and oxidative stress. It has been previously reported that decreased renal function may induce atherosclerosis^21,46^, and another study by Shim et al^22^. showed that POAG was significantly associated with increased systemic arterial stiffness, suggesting possible connection between the two conditions. Moreover, oxidative stress has been considered as one of well-known pathogenic mechanisms of OAG^23^, which also has been suggested as important underlying mechanism in CKD pathogenesis^24^. Also, low eGFR may reflect accumulation of reactive oxygen species^47^, and this may lead to increased susceptibility of glaucoma development by stimulating RGC apoptosis and glial activation in posterior segment of eye^48^. Such hypothesis may be supported by our findings that subjects suffering from more severe CKD condition showed increased HRs of developing OAG. Furthermore, previous studies showed significant association between elevated serum uric acid level and open-angle glaucoma^49–51^. Interestingly, serum uric acid level was reported to be commonly elevated in patients with CKD^52^, suggesting possible connection between the two diseases. However, further large scale prospective studies are needed to exactly evaluate these relationships.

Several previous cross-sectional population-based studies have demonstrated an association between lower eGFR and POAG^31,34^, but whether a causal relationship was present is uncertain. Our study, using a longitudinal population-based cohort design, showed an increased risk of OAG in CKD patients with advanced disease. We found that patients in advanced CKD group (CKD stages 3–5) had a significantly increased risk of OAG compared to mild to moderate CKD group (CKD stages 1–2). Although eGFR is the standard for staging CKD, the results from this study suggests that lower eGFR is an independent risk factor for OAG in CKD patients. Further studies should identify the pathogenetic mechanism underlying this association.

Our study has several limitations that should be considered when interpreting the results. First, the diagnoses of OAG, CKD, and other comorbidities were based entirely on KCD codes, which may be less accurate than the results of standard diagnostic tests performed in a real clinic. In this study, however, we included only OAG patients who met all three inclusion criteria: having the KCD code for an OAG diagnosis; having the code for visual field tests; and having a prescription for anti-glaucoma eye drops. As a result, the selection of OAG subjects used in the statistical analyses may have had high specificity. Second, the data used in our study were based only on patients who had visited a hospital or clinic during the study period. Therefore, the data excluded asymptomatic patients and those who had not visited hospitals for personal or economic reasons. This may have caused selection bias, leading to an underestimation of the true prevalence or incidence of OAG and CKD. However, the effects of any such bias or underestimation is thought to be limited; our government-run healthcare system includes almost the entire population of the country and is reasonably low-cost, making healthcare highly accessible.

In conclusion, our 12-year nationwide cohort study demonstrated that CKD patients have a higher risk of OAG than those without CKD. Moreover, more severe disease is associated with a significant increase in the risk of OAG. This indicates that lower eGFR is associated with OAG. The results of this study further support eye screening in CKD patients for early detection and treatment of OAG.

## Methods and materials

### Database and study sample

Our study used data from the NHIS-NSC 2002–2015. The Korean National Health Insurance Service (KNHIS) is a mandatory single-payer health insurance system in which all Koreans are enrolled. The National Health Information Database (NHID) developed by the KNHIS contains socioeconomic data (sex, age, income (insurance premium), etc.) and medical records. The NHIS-NSC sampled 1,103,302 of 46,605,433 individuals in the NHID in 2002 and followed them until 2015. The study design was approved by the Institutional Review Board of Soonchunhyang University Bucheon Hospital, Gyeonggi-do, Korea (IRB No. 2019-12-001–001), and the need for written informed consent was waived due to its minimal risk involved in the retrospective analysis. This study adhered to the tenets of the Declaration of Helsinki.

### Selection of patients and controls

The CKD cases were selected using the Korean Classification of Diseases (KCD) code N18. The OAG cases were defined as patients diagnosed with OAG (KCD code H401) and had one or more visit to an ophthalmologist after the index date. We included only newly diagnosed CKD patients by excluding those who had been diagnosed with CKD and had used medical services for the condition in 2002. Patients diagnosed with CKD from 2003 to 2008 were included. Patients who developed OAG before a diagnosis of CKD were excluded.

To analyze the effect of CKD on the risk of OAG, a control group was selected using propensity scores with nearest neighbor matching at a 1:5 ratio of CKD to non-CKD subjects. Ultimately, 25,560 subjects were included: 4260 in the CKD group and 21,300 in the non-CKD comparison group. These groups included 1318 OAG cases. Propensity scores were calculated using socioeconomic parameters, including age group (≤ 49, 50–59, 60–69, 70–79, ≥ 80), sex, household income percentile (≤ 30th, 31st–70th, ≥ 71st percentile according to insurance premium), residential area (urban included cities including metropolitan cities and rural included all other areas), and Charlson comorbidity index (CCI) scores (< 3, ≥ 3).

### Outcomes and comorbidities

We compared the incidence of OAG in the matched populations. CKD was the main outcome in the analysis. Subjects with CKD were divided into two groups based on CKD severity (mild to moderate CKD: CKD stages 1–3; advanced CKD: CKD stages 4–5). Socioeconomic variables and comorbid conditions were selected as confounding factors. Socioeconomic variables were age, sex, household income, residential area, and CCI. Comorbid conditions included hypertension (KCD codes I10–I15), diabetes mellitus (KCD codes E10–E14), hyperlipidemia (KCD codes E78.0–E78.5), and ischemic stroke (KCD code I61).

### Statistical analysis

The chi-square test was used to compare the groups. Multivariate Cox proportional hazard regression analysis was performed to calculate HRs with 95% confidence intervals (CI) for estimating the incidence of OAG in CKD patients. Survival curves for OAG were generated. All statistical analyses were conducted using SAS 7.3 and STATA 16.
